# Comparison among perfect-C®, zero-P®, and plates with a cage in single-level cervical degenerative disc disease

**DOI:** 10.1186/s12891-018-1950-9

**Published:** 2018-01-25

**Authors:** Sung Hyun Noh, Ho Yeol Zhang

**Affiliations:** 1 Department of Neurosurgery, National Health Insurance Service Ilsan Hospital, Goyang, South Korea; 2 Department of Neurosurgery, National Health Insurance Service Ilsan Hospital, Yonsei University College of Medicine, Goyang, South Korea

**Keywords:** Anterior cervical discectomy and fusion, Intervertebral fusion device, Heterotopic ossification, Subsidence

## Abstract

**Background:**

We intended to analyze the efficacy of a new integrated cage and plate device called Perfect-C for anterior cervical discectomy and fusion (ACDF) to cure single-level cervical degenerative disc disease.

**Methods:**

We enrolled 148 patients who were subjected to single-level ACDF with one of the following three surgical devices: a Perfect-C implant (41 patients), a Zero-P implant (36 patients), or a titanium plate with a polyetheretherketone (PEEK) cage (71 patients). We conducted a retrospective study to compare the clinical and radiological results among the three groups.

**Results:**

The length of the operation, intraoperative blood loss, and duration of hospitalization were significantly lower in the Perfect-C group than in the Zero-P and plate-with-cage groups (*P* < 0.05). At the last follow-up visit, heterotopic ossification (HO) was not observed in any cases (0%) in the Perfect-C and Zero-P groups but was noted in 21 cases (30%) in the plate-with-cage group. The cephalad and caudal plate-to-disc distance (PDD) and the cephalad and caudal PDD/anterior body height (ABH) were significantly greater in the Perfect-C and Zero-P groups than in the plate-with-cage group (*P* < 0.05). Subsidence occurred in five cases (14%) in the Perfect-C group, in nine cases (25%) in the Zero-P group, and in 15 cases (21%) in the plate-with-cage group. Fusion occurred in 37 cases (90%) in the Perfect-C group, in 31 cases (86%) in the Zero-P group, and in 68 cases (95%) in the plate-with-cage group.

**Conclusions:**

The Perfect-C, Zero-P, and plate-with-cage devices are effective for treating single-level cervical degenerative disc disease. However, the Perfect-C implant has many advantages over both the Zero-P implant and conventional plate-cage treatments. The Perfect-C implant was associated with shorter operation times and hospitalization durations, less blood loss, and lower subsidence rates compared with the Zero-P implant or the titanium plate with a PEEK cage.

## Background

Anterior cervical discectomy and fusion (ACDF) is regarded as the proper surgical treatment for symptomatic cervical degenerative disc disease of patients when conservative therapy is not effective. In 1958, Smith [1] and Cloward [2] initially introduced ACDF, which provided both neural decompression and spine stability. Long-term follow-up revealed that ACDF is an effective method but that up to 25% of patients may develop radiculopathic or myelopathic symptoms [3].

**Fig. 1:**
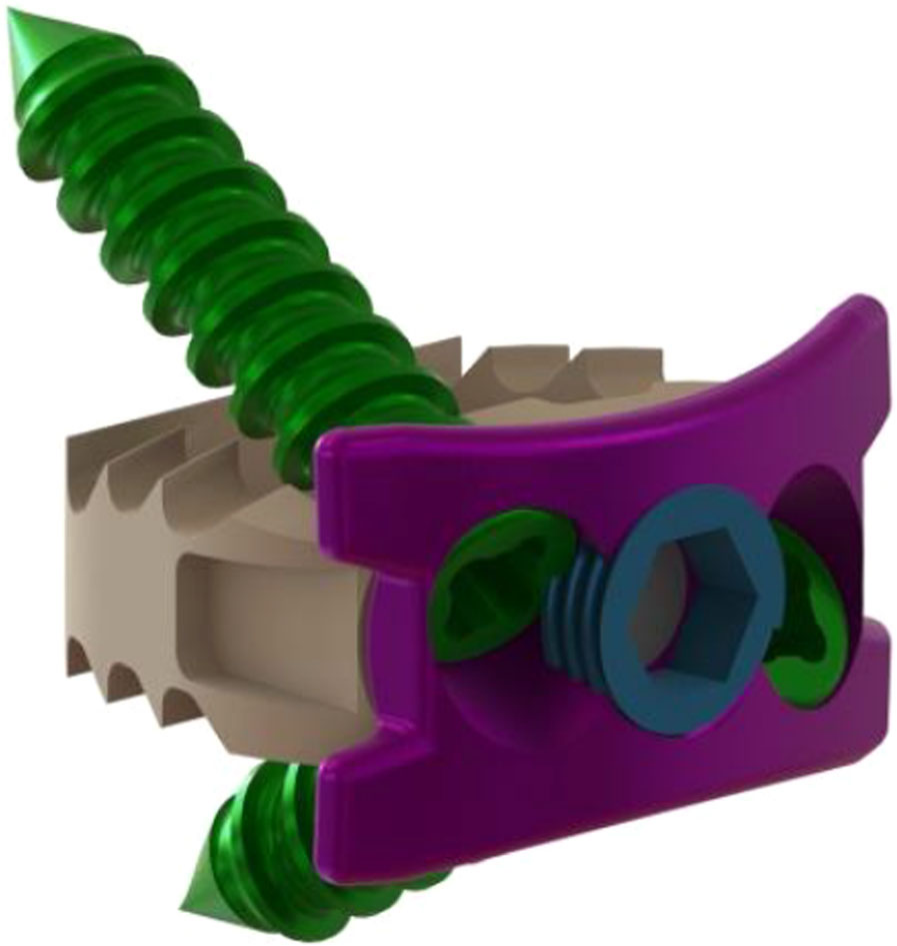
Photograph of a Perfect-C implant (Seohancare, Seoul, South of Korea)

**Fig. 2:**
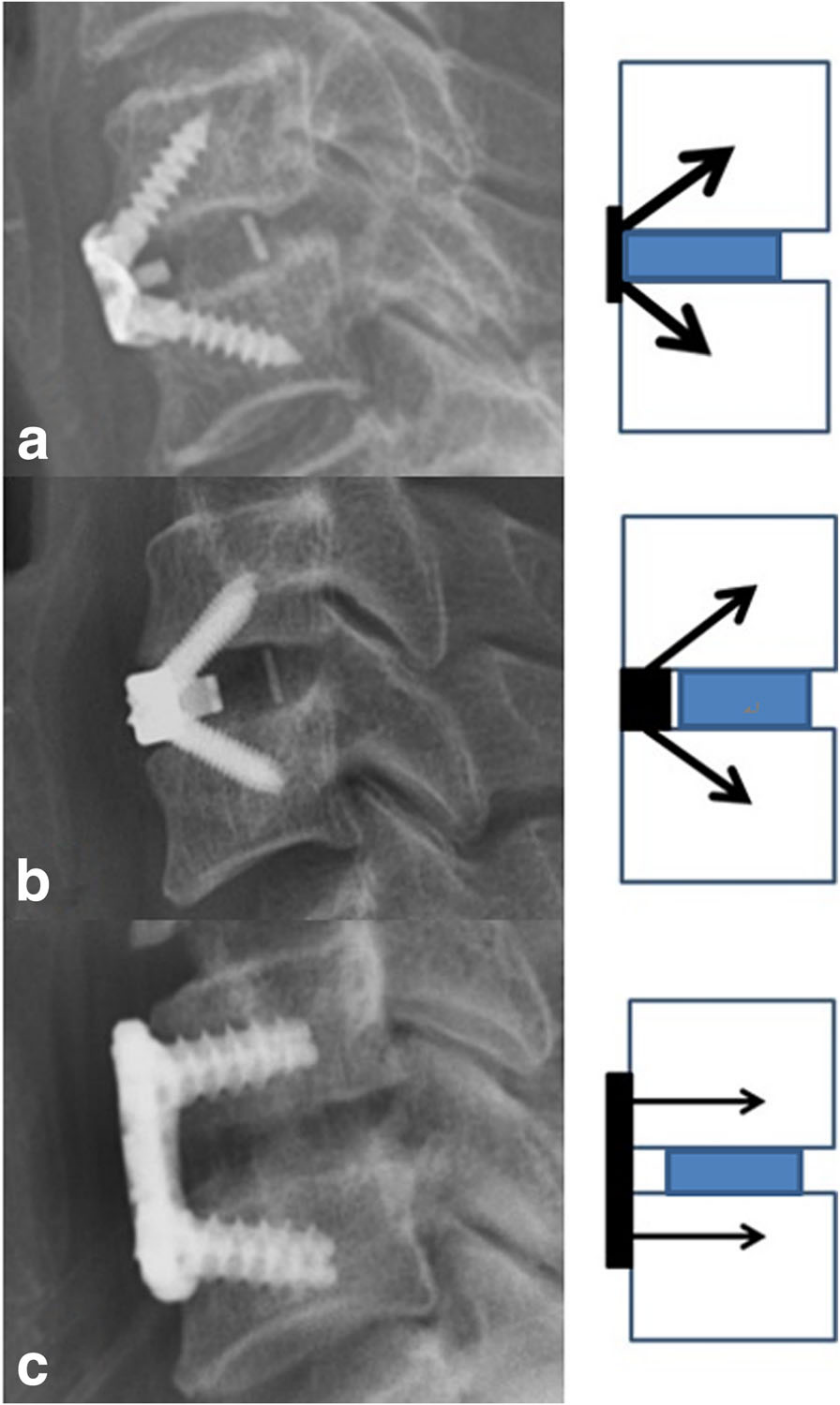
**a**. The Perfect-C enters the cancellous bone of the vertebral body through the cortical bone, which is similar to the plate with cage system. **b**. The Zero-P enters the cancellous bone of the vertebral body through the endplate. **c**. The plate with cage system directly enters the cortical bone

Titanium plates and cages support many advantages over other approaches for ACDF, including a higher fusion rate, disc height restoration, better cervical lordosis and alignment [4]. However, complications such as screw or plate displacement, soft tissue damage, esophageal perforation, and dysphasia have been reported [5]. Of particular concern, anterior osteophyte development and anterior longitudinal ligament ossification have been appeared after anterior cervical fusion [6]. In 2009, a new integrated cage and plate device (Perfect-C, Seohancare, Korea) was developed, which integrates the benefits of an anterior cervical plate and a fusion cage to prevent complications such as heterotopic ossification (HO) (Fig. 1). A report outlining the potential applications of the Perfect-C was later published in 2011 [7]. In fact, the Perfect-C was developed before introduction of the Zero-P in Korea in 2013. The Perfect-C is composed of a polyetheretherketone (PEEK) cage with two screws, which enter the cancellous bone of the vertebral body through the corner between the cortical bone and the endplate (Fig. 2a). In contrast, the screws of the Zero-P implant enter the cancellous bone of the vertebral body through the endplate, and the screws of plate-with-cage implants directly enter the cortical bone (Fig. 2b, c). Minimizing the contact between the plate and anterior soft tissue creates less HO and is associated with less dysphagia. In general, the Zero-profile integrated plate and spacer device (Zero-P, Synthes, Oberdorf, Switzerland) is employed for the treatment of cervical degenerative disc disease and disc herniation. The Zero-P has many advantages that are similar to those of the Perfect-C. We compared the clinical and radiological results of patients who received a Perfect-C implant, a Zero-P implant, or a titanium plate with a PEEK cage for the treatment of cervical degenerative disc disease.

## Methods

### Patients

The inclusion criteria were set as follows: 1) symptoms of cervical radiculopathy and/or myelopathy; 2) failure of conservative treatments, including medication, physical therapy, and injection therapy lasting more than 6 weeks; and 3) cervical spine X-ray, computed tomography (CT), and/or magnetic resonance imaging (MRI) indicating intervertebral disc degeneration. The exclusion criteria included the following: 1) the presence of dysphagia before surgery; 2) patients who had undergone previous cervical spine surgery or had a history of other cervical spine disease including fractures, tumors, etc.; and 3) patients who underwent simultaneous anterior and posterior cervical operations.

One hundred forty-eight patients with single-level degenerative cervical disc disease who were subjected to ACDF between January 2012 and March 2016 at the authors’ institution and conformed to the inclusion and exclusion criteria were examined in this study. The patients were divided into three groups according to the implants used during ACDF: 41 patients received a Perfect-C implant, 36 patients received a Zero-P implant, and 71 patients received titanium plates and a PEEK cage (Plate-Cage group).

### Perfect-C integrated plate and spacer device

In 2012, the Perfect-C integrated plate and spacer device was approved by the Korean Ministry of Food and Drug Safety as a substitute for the conventional dividing interbody spacer and plate device used for ACDF. Perfect-C forms an interbody spacer with the front plate, at least protruding out of the disk space like the front cervical plate. The PEEK interbody spacer incorporates a radiopaque indicator used for visualization by X-ray. Two 3.5~ 3.9-mm-diameter screws are inserted within the plate at a 40 ± 10° cranial or caudal angle. In contrast to the Zero-P implant, which is not placed in front of the anterior column in the lateral view, the Perfect-C implant is placed 1 mm ahead of the anterior column in the lateral view, and the screw was designed to penetrate the vertebral body in the corner of the cortical bone and endplate.

### Surgical procedure

The patients were placed under general anesthesia in the supine position. The operative method was the standard Smith-Robinson technique. After identification and exposure of the relevant vertebral levels corresponding to the compressive sources, a discectomy was conducted, and a high-speed burr was used to eliminate the anterior and posterior bony spurs and the cartilaginous endplate. The endplate cartilage was removed with a curette and diamond drill, and the bony endplate was maintained as much as possible to avoid cage subsidence. The posterior longitudinal ligament was dissected. Dural and neural decompression was achieved after compressive sources were eliminated. In the Perfect-C and Zero-P groups, trial spacers decide the relevant size of the cage. Local osteophytes were eliminated. First, 1 cm^3^ of recombinant human bone morphogenetic protein (Rafugen DBM, Cellumed, Seoul, Korea) was delivered inside the PEEK cage, and the cage was then placed with an impactor into the center of the disc space. After implantation of the cage, two screws for the Perfect-C device or four screws for the Zero-P device were located in both sides of vertebrae through the anterior portion of the cage to support stabilization. In the Plate-Cage group, the size of the PEEK cage was determined by both preoperative formatting and intraoperative estimates using a trial cage [8]. The PEEK cage was prepared with 1 cm^3^ of Rafugen DBM after eliminating osteophytes, and the cage was inserted into the disc space [8]. And then screws were employed to place the anterior cervical plate [8]. The patients were allowed to sit upright 12 hours after the operation, and they could walk on the first day with a neck collar. The clinical and radiographic results analyses were conducted 5 days postoperatively by an unaffiliated observer.

### Clinical outcome assessment

The clinical outcomes were estimated using the neck disability index (NDI) score and a visual analog scale (VAS) preoperatively and at the last follow-up. The length of the operation, intraoperative blood loss, and intraoperative complications such as spinal cord injury, vessel injury, esophageal damage, superior laryngeal nerve injury, and recurrent laryngeal nerve injury were reviewed and analyzed retrospectively. The incidence of dysphagia was detected using the system defined by Bazaz [9].

### Radiological evaluation

**Fig. 3:**
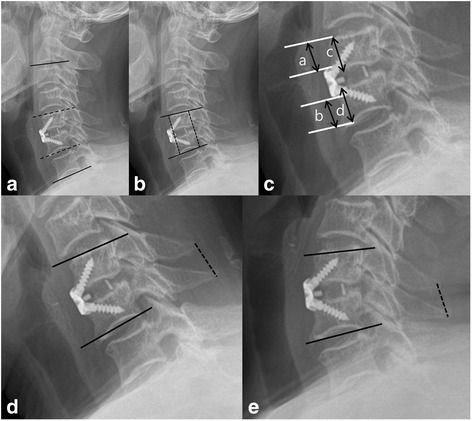
**a** Cervical alignment was formed by the lines along the lower endplate of C2 to the lower endplate of C7 in the neutral position. The segmental angle was assessed using the Cobb angle of the vertebral bodies adjacent to the involved disc. **b** The disk height was the distance from the lower endplate of the cephalad vertebral body to the upper endplate of the caudal vertebral body of the fusion segment was measured. And disc height was measured as the average value of the height of the anterior portion and posterior. **c** The cephalad and caudal plate-to-disc distance (PDD) and the PDD/anterior body height (ABH) ratio were measured. **d** The segmental angle was measured using the Cobb angle on flexion X-ray. The interspinous distance was measured on flexion X-ray across the fusion segment. **e** The segmental angle was measured using the Cobb angle on extension X-ray. The interspinous distance was measured on extension X-ray across the fusion segment

The preoperative study included plain radiographs, CT, and MRI. Plain radiological studies of the cervical spine were also performed immediately after the operation and at the last follow-up visit for all patients. Cervical alignment was evaluated using the Cobb angle of C2–C7 according to the method described by Borden [10]; this angle is measured by the lines along the lower endplate of C2 to the lower endplate of C7 in the neutral position. The segmental angle was assessed using the Cobb angle of the vertebral bodies adjacent to the involved disc (Fig. 3a). The distance from the lower endplate of the cephalad vertebral body to the upper endplate of the caudal vertebral body of the fusion segment was measured. And disc height was measured as the average value of the height of the anterior portion and posterior portion (Fig. 3b) [11]. Subsidence was defined by calculating the distance from the superior endplate of the upper vertebral body to the inferior endplate of the lower vertebral body at the level of the operation [12]. Subsidence calculations were performed from the anterior and posterior borders of the vertebral bodies [12]. Subsidence was explained as a decline in the height of the surgical segment greater than 3 mm between the images obtained immediately after the operation and those obtained at the last follow-up. The prevertebral soft-tissue thickness was decided by evaluating the thickness of soft tissue from the anterior side of the C3–7 vertebrae to the posterior side of the trachea [12]. The cephalad plate-to-disc distance (PDD) and caudal plate-to-disc distance (PDD) were also measured, and the PDD/ABH (PDD to anterior body height) ratio was then determined (Fig. 3c). Radiological fusion was defined that there was ≤ 2° motion and/or ≤ 2 mm of motion of the interspinous distance on flexion–extension x-rays (Fig. 3d, E) [13].

### Statistical analysis

The findings are presented as the mean values ± standard deviation (SD) or counts, as indicated. For the three groups, one-way analysis of variance (ANOVA) and chi-squared tests were used for comparisons, as appropriate. The results were adjusted for age and sex. We calculated adjusted *P*-values using the Bonferroni method for multiple comparisons. A P-value < 0.05 was considered statistical significance. All statistical analyses were performed using SPSS (version 23.0, SPSS, Chicago, IL, USA).

## Results

### Patient demographics (Table 1)

One hundred forty-eight patients underwent ACDF at the author’s institution. Table 1 shows the detailed demographics of the three groups of patients, which are comparable. This study included 88 (59.5%) male and 60 (40.5%) female patients. The patients’ ages ranged from 33 to 87 years old (average age, 56.5 ± 10.93 years old), and the patients were followed for an average of 32.7 ± 17.5 months.

**Table 1:** Patient demographics

	Perfect-C (*n* = 41)	Zero-P (*n* = 36)	Plate-Cage (*n* = 71)	*P*-value
Sex				
Female	18	11	31	
Male	23	25	40	0.374
Mean age	57.46 ± 11.35	55.64 ± 10.31	55.06 ± 11.13	0.533

### Comparison of intraoperative blood loss, operation time, days of hospitalization, and clinical parameters (Table 2)

Intraoperative blood loss, operation time, days of hospitalization, and clinical parameters of the three groups are shown in Table 2. The operation time, intraoperative blood loss, and days of hospitalization were lower in the Perfect-C group than in the Zero-P and Plate-Cage groups (*P* < 0.05). The NDI scores and VAS were improved at the last follow-up (*P* < 0.0001). One patient in both the Perfect-C group and the Zero-P group exhibited dysphagia 1 month after the operation, whereas 8 patients in the Plate-Cage group exhibited dysphagia 1 month after the operation. There were no significant differences in the occurrence of dysphagia among the three groups (*P* > 0.05).

**Table 2:** Comparisons of intraoperative blood loss, operative time, and days of hospitalization, and clinical parameters

	Perfect-C (*n* = 41)	Zero-P (*n* = 36)	Plate-Cage (*n* = 71)	*P*-value
Intraoperative blood loss (ml)	69.51 ± 22.91	74.44 ± 17.15	93.66 ± 45.65	0.001*
Operation time (min)	107.81 ± 17.96	113.33 ± 19.12	127.96 ± 30.37	< 0.001*
Duration of hospitalization (day)	6.35 ± 1.14	6.47 ± 2.55	8.93 ± 5.17	< 0.001*
NDI scores				
Preoperation	38.19 ± 3.84	38.47 ± 1.76	38.84 ± 2.13	0.443
last follow-up	14.34 ± 2.02^#^	14.45 ± 2.39^#^	14.86 ± 2.23^#^	0.381
VAS				
Preoperation	8.09 ± 0.74	8.36 ± 0.75	8.47 ± 0.7	0.078
last follow-up	2.68 ± 0.96^#^	2.89 ± 0.95^#^	3.16 ± 0.97^#^	0.034*
Dysphasia				
Postoperative 1 month	1 (2%)	1 (3%)	8 (11%)	0.262
last follow-up	0	0	1 (1%)	0.108

*NDI* neck disability index, *VAS* visual analog scale

*P < 0.05, comparison among the Perfect-C, Zero-P, and Plate-Cage groups

^#^*P* < 0.05, comparison with the preoperative value

### Comparisons of the radiologic parameters (Table 3)

The cervical alignment, segmental angle, intervertebral height, incidence of subsidence, fusion rate, and prevertebral soft-tissue thickness among the three groups are shown in Table 3. In addition, the PDD/ABH ratio was calculated using postoperative lateral radiographs to identify the relevance of HO. The three groups’ cervical alignment, segmental angle, and intervertebral height at the last follow-up were markedly improved compared with those at the preoperative assessment (*P* < 0.05). In terms of the prevertebral soft-tissue thickness, the Perfect-C and Zero-P groups showed recovery at the last follow-up (*P* < 0.0001). In addition, there were significant differences in the PDD, PDD/ABH and incidence of HO among the three groups (P < 0.0001).

**Table 3:** Comparisons of radiologic parameters

	Perfect-C (*n* = 41)	Zero-P (*n* = 36)	Plate-Cage (*n* = 71)	*P*-value
Cervical alignment (°)				
Preoperation	12.39 ± 6.78	12.49 ± 7.06	14.11 ± 9.24	0.457
Postoperation	17.35 ± 7.87^#^	16.47 ± 8.03^#^	15.28 ± 9.6^#^	0.471
Last follow-up	20.57 ± 8.54^#^	18.41 ± 8.11^#^	17.76 ± 9.85^#^	0.455
Segmental angle (°)				
Preoperation	5.81 ± 3.8	4.94 ± 3.89	4.59 ± 3.98	0.285
Postoperation	6.1 ± 4.49^#^	5.25 ± 3.15^#^	5.41 ± 3.07^#^	0.514
Last follow-up	6.59 ± 6.17^#^	5.34 ± 3.69^#^	5.34 ± 3.06^#^	0.294
Intervertebral height (mm)				
Preoperation	5.48 ± 1.48	5.93 ± 1.01	5.75 ± 1.21	0.284
Postoperation	7.81 ± 1.31^#^	7.56 ± 1.01^#^	7.62 ± 1.12^#^	0.58
Last follow-up	6.77 ± 1.49^#^	6.48 ± 1.17^#^	6.22 ± 1.14^#^	0.082
Subsidence	5 (14%)	9 (25%)	15 (21%)	0.546
Fusion	37 (90%)	31 (86%)	68 (95%)	0.284
Prevertebral soft-tissue				
Thickness (mm)				
Preoperation	15.27 ± 2.88	12.17 ± 3.32	13.15 ± 3.29	
Postoperation	18.59 ± 3.34^#^	15.4 ± 3.63^#^	18.97 ± 3.91^#^	
Last follow-up	14.91 ± 2.96^#^	11.86 ± 3.1^#^	15.01 ± 3.95^#^	
PDD (Cephalad, mm)	10.52 ± 2.12	13.72 ± 2.11	5.33 ± 2.09	< 0.001*
PDD (Caudal, mm)	11.83 ± 2.31	14.89 ± 1.58	6.03 ± 1.99	< 0.001*
PDD / ABH (Cephalad)	0.74 ± 0.09	1	0.38 ± 0.12	< 0.001*
PDD / ABH (Caudal)	0.77 ± 0.05	1	0.39 ± 0.11	< 0.001*
HO	0	0	21 (30%)	< 0.001*

*PDD* plate-to-disc distance, *ABH* anterior body heights, *HO* heterotopic ossification

*P < 0.05, comparison among the Perfect-C, Zero-P, and Plate-Cage groups

^#^*P* < 0.05, comparison with the preoperative value

## Discussion

ACDF is the most common method in degenerative disc disease of the cervical spine when conservative therapy has failed [12]. Many anterior cervical instruments have been introduced due to the continuous development of surgical methods and devices. The composition of an anterior cervical plate and an interbody cage in ACDF is possible to increase fusion rates compared with the ACDF without anterior plates [12, 14]. Numerous articles have reported the effective use of placing plates to prevent pseudoarthrosis, subsidence, and local kyphosis [15]. However, the addition of a plate may cause soft-tissue injury, dysphagia, plate fracture, and migration. [16]. The Perfect-C implant was made in 2009 before the Zero-P implant was introduced to Korea. The Perfect-C implant is a new integrated cage and plate device that combines the benefits of an anterior plate and a cage to help avoid complications such as HO and dysphasia. The Zero-P leads to good fusion rate and biomechanical stability and both the Zero-P and Perfect-C implants can be used to correct cervical kyphosis and improve cervical alignment [17]. Herein, we explain various aspects of the Perfect-C in comparison with the Zero-P and the plate-with-cage system.

The Zero-P device is relatively easy to use, and the mean length of the operation and amount of blood loss were previously shown to be lower than for Plate-Cage surgeries in one- and two-level procedures [17]. A significant decrease in intraoperative blood loss was confirmed for one-level surgery, and a significant decrease in the length of the operation was demonstrated for two-level surgery in patients treated using the Zero-P device [18]. Wang et al. [19] reported less intraoperative blood loss for two-level surgery with the Zero-P device because few steps are required to insert the Zero-P device due to its one-step locking system with simple placing of the cage and fixation of the screws. Clavenna et al. [20] reported that method of integrated plate with a cage permits a shorter operation with fewer surgical procedures as well as similar equipment stability compared with the conventional plate and cage.

In our present study, the length of the operation time and the amount of blood loss were lowest for the Perfect-C group, and these differences were statistically significant (Table 2). The surgical procedure for implantation of the Perfect-C is similar to that for implantation of the Zero-P. However, the Perfect-C device requires only two-screw fixation, while the Zero-P requires four-screw fixation. When implanting the Zero-P system, it is very difficult to achieve the optimal angle for inserting screws in the lower C3/4 and upper C6/7, particularly in patients with a short neck or a high sternum [16]. For this reason, the screws of the Zero-P form a bone wedge with a 40 **±** 5° cranial and caudal angle. In contrast, the screws of the Perfect-C form a bone wedge with a 40 **±** 10° cranial and caudal angle. We observed cases of C3/4 and C6/7 insertion in both groups. Decreases in intraoperative blood loss and the length of the operation can decrease the damage caused by surgery and reduce the risk of complications. These factors also contribute to the recovery of patients after operation, allowing them to obtain a better outcome, and may also influence the period of hospitalization. Supporting this possibility, the hospitalization duration was shortest in the Perfect-C group.

The patients in all three groups showed statistically significant reductions in NDI and VAS scores. Thus, ACDF with either the Perfect-C, the Zero-P, or a plate-with-cage implant can be considered an efficient treatment for cervical degenerative disc disease. However, dysphagia is a relatively common complication after ACDF. Although no definitive mechanism has been proven, swelling due to traction, hematoma, and nerve injury such as the pharyngeal nerve plexus and the hypoglossal nerve during operation are considered risk factors [15]. Duan et al. [18] reported that the incidences of dysphagia in patients administered the Zero-P device were significantly lower than those in patients using a plate and cage. This finding suggests that the occurrence of postoperative dysphagia is associated with the design and thickness of the plate [21]. Because the plate is located ahead of the vertebral body and behind the esophagus, it can annoy the esophagus and cause dysphagia. On the other hand, the Zero-P device reduces the development of dysphagia because it is located completely within the intervertebral space. The Zero-P is therefore less irritating to the esophagus and other prevertebral soft tissues. Miao [22] and Hofstetter [23] found significantly lower rates of dysphagia in patients treated with the Zero-P device compared to those treated by plating. In our study, the incidences of dysphagia in the Perfect-C and Zero-P groups were similar to or lower than that in the Plate-Cage group. Perfect-C comprises the interbody spacer with the front plate, at least protruding out of the disk space like the front cervical plate and to a lesser degree than anterior cervical plates. The device is placed 1 mm ahead of the anterior column in the lateral view. Therefore, the plate of the Perfect-C minimally irritates the esophagus and other prevertebral soft tissues.

**Fig. 4:**
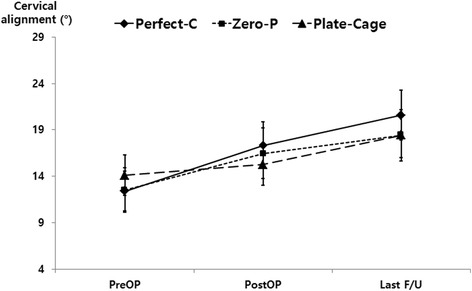
The cervical alignments of the three groups were significantly different between the pre- and postoperative findings. In addition, the findings obtained at the last follow-up visit were significantly improved compared with those obtained at the preoperative assessment (*P* < 0.05)

**Fig. 5:**
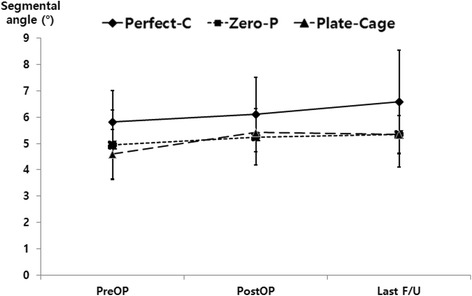
The segmental angles of the three groups were significantly different between the pre- and postoperative findings. In addition, the findings obtained at the last follow-up visit were significantly improved compared with those obtained at the preoperative assessment (*P* < 0.05)

**Fig. 6:**
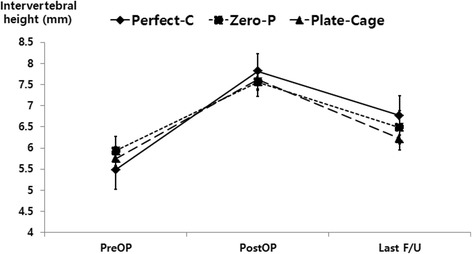
The intervertebral heights of the three groups were significantly different between the pre- and postoperative findings. In addition, the findings obtained at the last follow-up visit were significantly improved compared with those obtained at the preoperative assessment (*P* < 0.05)

Preservation of normal lordotic alignment is necessary for stable motion and function in the cervical spine [4]. Loss of normal alignment after ACDF surgery may lead to postoperative axial pain, aggravation of neurologic deficits, and delay of the patient’s functional recovery [4]. Furthermore, loss of normal alignment could be associated with increased adjacent segment degeneration during long-term follow-up [24]. In our study, the cervical alignment, segmental angle, and intervertebral height of three groups at the last follow-up visit showed obvious improvements compared with the preoperative status. There were significant differences between the pre- and postoperative findings and between the findings obtained preoperatively and at the last follow-up visit (*P* < 0.05) (Figs. 4, 5, 6).

The reported rates of subsidence after ACDF vary from 5.4% to 55.6% according to the surgical method [12]. It is currently controversial regarding whether the clinical outcome of surgery is associated with subsidence. However, subsidence causes secondary kyphotic changes, which are risk factors for progressive degenerative changes in adjacent levels. Lee et al. [25] reported that the subsidence rate with the Zero-P (58.6%) was higher than that with PEEK cage and plating (38.5%). It means that plates maintain anterior disc height to prevent subsidence. In another study, Lee et al. [26] reported that the subsidence rate of the Zero-P device (21.7%) was higher than the plate-with-cage (11.1%) On the other hand, Scholz et al. [27] reported that subsidence did not occur in patients treated with the Zero-P device in follow-up lasting only 6 months. Our present results showed that the subsidence rate of the Perfect-C (14%) group was lower than that of the Zero-P (25%) group and the Plate-Cage (21%) group at the last follow-up.

One reason for the minimal subsidence of the Perfect-C device is that the screws of the Perfect-C enter the cancellous bone of the vertebral body through the anterior corner of the vertebral body and endplate (Fig. 2a). Two large diameter screws have a high resistance to pull-out force because this area is the hardest and thickest portion of the endplate [28]. Another reason is that the modulus of the PEEK cage of the Perfect-C device is similar to that of the bone located at the thick anterior endplate. On the other hand, the screws of the Zero-P device enter the cancellous bone of the vertebral body through the endplate (Fig. 2b). Furthermore, the titanium plate of the Zero-P is stronger than the modulus of the bone that contacts the hard anterior endplate. In addition, the PEEK cage is located at the thinnest area of the main part of the endplate; thus, subsidence can occur more easily and is likely the reason for the differences between the Perfect-C and Zero-P groups. The screws of the plate with cage directly enter the cortical bone (Fig. 2c). In a study by Gercek et al., the subsidence occurred in five (55.6%) of nine patients, but no association between subsidence and clinical outcomes was observed [29]. The incidence of subsidence can presumably be affected by various factors, such as the size, position, and contact area of the cage as well as the bone quality and the conducted distraction during the operation. Therefore, further study using a larger number of patients with a longer follow-up period will be necessary to draw a conclusion regarding the best approach and most important clinical and radiographic outcomes.

The radiographic fusion rate of the ACDF group was 94.3% in 221 patients with a median follow-up of more than 3 years in a randomized controlled clinical trial comparing standard ACDF [30]. Similarly, Coric et al. [31] reported a 97% fusion rate after ACDF surgery with an average follow-up of 6 years. In our study, the fusion rate was 90% in the Perfect-C group, 86% in the Zero-P group, and 95% in the Plate-Cage group at the last follow-up visit. However, long-term follow-up is needed to accurately assess the fusion rates.

The prevertebral soft-tissue thickness showed obvious improvement in all three groups at the last follow-up visit compared with the findings obtained immediately after the operation. In addition, there was a statistically significant difference between the pre- and immediate postoperative findings and between the pre- and last follow-up findings among the three groups (*P* < 0.0001) (Table 3). However, we did not find any significant relationship between prevertebral soft-tissue thickness and dysphagia after operation. This means that prevertebral soft-tissue injury may not be associated with dysphagia, and the mechanism underlying the development of dysphagia after ACDF remains unclear. During the postoperative period, esophageal injury, recurrent laryngeal nerve palsy, adhesions, and instrument failure may contribute to dysphagia [12]. Thus, minimizing the retraction pressure may lead to prevent the esophagus damage [32].

Plating in ACDF is associated with the occurrence of HO. Park et al. [33] reported that the prevalence of HO significantly increased when the plate is located within 5 mm from near the disc. Garrido et al. [34] also reported that HO occurred in 50% of patients when the plate-to-disc distance is within 5 mm. Yang et al. [35] reported that HO was not common after ACDF without plates and occurred in only 5.5% of patients compared to the rate of 45.4% following ACDF with plating. The Perfect-C and Zero-P devices were designed to decrease the incidence of HO. Yang et al. [16] reported that the HO incidence of Zero-P was only 1.6%, but the that of the plate and cage was 18.8%. In our study, we measured the cephalad PDD, the caudal PDD, and the PDD/ABH ratio and found significant differences in these parameters among the three groups (Table 3). The rates of HO in our groups were 0% (Perfect-C and Zero-P groups) and 30% (Plate-with-Cage group) at the last follow-up visit, which corresponded to significant differences among groups. Although the mechanism of HO is known, the association of anterior structures, such as the anterior longitudinal ligament or anterior fibers of the annulus fibrosis, and other technical ad biomechanical considerations related to plating could be regarded as contributing factors [15].

### Limitations of this study

Our study was characterized by certain limitations. Because our study did not have a randomized controlled design, we could not completely control the possibility of selection bias. Additionally, because our study size was small, there was a limitation in the number of comparisons that could be made between groups for several factors that are known to affect prognosis. However, the results of this study suggest that the Perfect-C device is safe and effective for the treatment of single-level cervical degenerative disc disease.

## Conclusion

The results of the present study comparing the Perfect-C, Zero-P, and plate-with-cage approaches showed that the Perfect-C device is as effective as the Zero-P and plate-with-cage devices for the treatment of cervical degenerative disc disease. The Perfect-C device was associated with a simple operation, low blood loss, a short operation time, and low incidences of dysphagia, subsidence, and HO. However, the follow-up period in our study was short, and the number of patients was small. Thus, a study including a larger number of patients and long-term follow-up is necessary to accurately determine the outcomes of this device compared with the Zero-P device and the conventional plate-and-cage approach.

## Abbreviations

ABH: Anterior body heights
ACDF: Anterior cervical discectomy and fusion
CT: Computed tomography
HO: Heterotopic ossification
MRI: Magnetic resonance image
NDI: Neck Disability Index
PDD: Plate-to-disc distance
PEEK: Polyetheretherketone
VAS: Visual analog scale
